# Dravet Syndrome Patient-Derived Neural Cells Present Altered Levels of Potassium, Copper, and Zinc

**DOI:** 10.1021/acschemneuro.5c00898

**Published:** 2025-12-24

**Authors:** Mariana P. Stelling, Rafaela C. Sartore, Gabriela L. Vitória, Sylvie Devalle, Marília Z. P. Guimarães, Stevens K. Rehen, Simone C. Cardoso

**Affiliations:** † Federal Institute of Education, Science and Technology of Rio de Janeiro, Rio de Janeiro 20270-021, Brazil; ‡ National Institute of Traumatology and Orthopaedics, Rio de Janeiro 20940-070, Brazil; § D’Or Institute for Research and Education, Rio de Janeiro 22281-100, Brazil; ∥ Institute of Biomedical Sciences, D’Or Institute for Research and Education, Federal University of Rio de Janeiro, Rio de Janeiro 21941-902, Brazil; ⊥ D’Or Institute for Research and Education and Institute of Biomedical Sciences, 519983 Federal University of Rio de Janeiro, Rio de Janeiro 21941-902, Brazil; # Physics Institute, Federal University of Rio de Janeiro, Rio de Janeiro 21941-909, Brazil

**Keywords:** Dravet syndrome (DS), Nav1.1 loss-of-function, elemental imbalance, iPS cells, synchrotron X-ray radiation

## Abstract

Dravet syndrome (DS), also known as severe myoclonic epilepsy of infancy (SMEI), is an intractable epilepsy syndrome. Most cases are associated with mutations in the SCN1A gene, which is responsible for the expression of the sodium voltage-gated channel alpha subunit 1, Nav1.1. These mutations lead to altered neuronal firing and a state of hyperexcitability. DS has been studied using patient samples, animal models, and more recently, iPS cells derived from DS patients. In this work, we sought to understand the impact that Nav1.1 loss-of-function has on the elementary chemical constitution of DS patient-derived neural cells. iPS cells from DS patients and controls were differentiated into neural-induced spheroids, and synchrotron X-ray radiation was used to assess alterations in their elemental concentration. We observed that DS-derived neural cells present elevated levels of potassium (K), copper (Cu), and zinc (Zn). These findings suggest that an elemental imbalance may be involved in the pathogenesis of DS, as higher levels of K, Cu, and Zn have been implicated in seizure episodes and epilepsy. We conclude that modeling DS using cell reprogramming is a relevant approach to understanding the basic mechanisms involved in this disease and perhaps provide novel treatment strategies.

## Graphical abstract

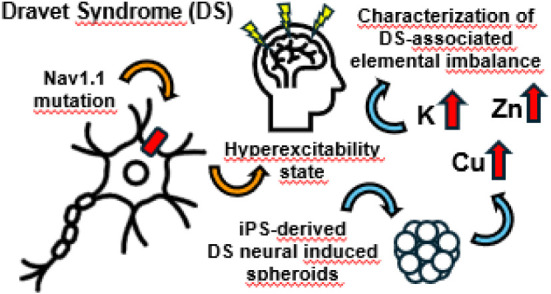

## 1 Introduction

Dravet syndrome (DS) is a genetic pediatric epileptic syndrome also known as severe myoclonic epilepsy of infancy (SMEI), which begins in early childhood and results in a high mortality rate (up to 15%) and severe cognitive and neurological sequelae,^1^ in addition to motor impairment.^2^ Most cases are associated with mutations in the α-subunit of the neuronal sodium channel Nav1.1 gene (SCN1A).^3,4^ Previous studies have shown that Nav1.1 channels are mostly expressed in the central nervous system, especially in GABAergic hippocampal interneurons.^5–7^ Currently, advanced treatments using the CRISPR technique or targeting the splicing of the SCN1A gene are being considered for more effective treatments, because to this day the aim is focused in controlling seizures.^2,8^ Loss-of-function mutations in Nav1.1 channels lead to altered neuronal firing,^9^ culminating in a state of hyperexcitability. Abnormal neuronal firing may arise from, or be worsened by, mutated ion channels, such as Nav1.1 in DS. One mutated channel not only affects its own permeant ion, but also, overall elemental distribution, as neural tissue ionic equilibrium is finely controlled.^10,11^ Different types of epilepsy are profoundly associated with elemental imbalance,^12^ since changes in the concentration of one ion can affect others.

There is sparse data regarding elemental distribution in DS tissues, nevertheless, the impact of changes in a few elements such as calcium, zinc, copper, and magnesium has been described in other types of epileptic syndromes.^13,14^ Most of that evidence is based on serum analyses of patients under treatment, which confounds data interpretation. Therefore, it is important to develop cellular models not only to test novel drugs and treatment strategies, but also to emulate tissues under untreated conditions. Our group aimed to specifically investigate elemental distribution in DS using induced pluripotent stem cells differentiated into neural cells. This model may be used for recapitulation of human neural development by an assortment of protocols, such as embryoid body^15^ and neurosphere formation,^16^ and cerebral organoid production,^17^ all with minimal, or absence of, interference from drug treatments or environmental factors.

Here we aimed to unveil the basic, cell-related elemental changes that may occur in DS, bringing to light new perspectives on such devastating disease.

## 2 Materials and Methods

### 2.1 Cell Lines, Embryoid Body Formation, and Neural Induction

The study was approved by the Institutional Research Ethics Committee of the D’Or Institute for Research and Education (60944916.5.0000.5249 and 31239914.7.0000.5249). Informed consent was obtained from Dravet syndrome (DS) patients and/or their legal tutors. Urine samples were donated by patients clinically diagnosed with DS. Cells collected from urine samples were cultured and reprogrammed as previously described.^18^

Control iPS cells were obtained from different sources as stated in the work by Casas and colleagues,^19^ lines CF1 and CF2 were reprogrammed in-house, while GM23279*A is commercially available (Coriell Institute for Medical Research, USA). DS iPS cells named: DRVT1, DRVT2 and DRVT3 were also reprogrammed in-house and cultured in mTeSR1 medium (Stemcell Technologies, USA) on a Matrigel-coated surface (BD Biosciences, USA). The medium was changed daily. Colonies were manually passaged and maintained at 37 °C in humidified air with 5% CO_2_.

Confluent iPS cells were dissociated to a single-cell suspension by enzymatic treatment with TrypLE Express (Invitrogen, USA) and cultured in 60 mm nonadherent plates in the following medium: high-glucose DMEM and F12 1:1 (v:v, 11330057, Thermo Fisher Scientific, USA) supplemented with 15% KSR (10828–028, Thermo Fisher Scientific, USA), 200 mM Glutamax (35050061, Thermo Fisher Scientific, USA), 55 mM 2-mercaptoethanol (21985023, Thermo Fisher Scientific, USA), and 100 μM nonessential amino acids (11140–050, Thermo Fisher Scientific, USA). Embryoid bodies (EBs) were cultured in this condition for 7 days and the medium was changed twice per week.

In order to reach a neural induced spheroid profile, at day 7 EB medium was replaced with one consisting of high-glucose DMEM and F12 (1:1), 1 mg/mL bovine heparin (Cristália, Brazil), 1% nonessential amino acids (11140–050, Thermo Fisher Scientific, USA), 1% N2 supplement (17502001, Thermo Fisher Scientific, USA) and 0.5 ng/mL FGF-2 (PHG0261, Thermo Fisher Scientific, USA). Medium was changed every other day. Upon completion of the differentiation protocol, samples were collected for immunofluorescence staining, RT-PCR, and X-ray Fluorescence (XRF) multi-elemental quantification and mapping.

### 2.2 Immunofluorescence

Neural induced spheroids were fixed in 4% paraformaldehyde for 10 min, sequentially incubated in sucrose solutions (10%, 20%, and 30%), embedded in optimal cutting temperature compound (OCT), frozen in liquid nitrogen and stored at −70 °C. Samples were cryosectioned (Leica, Germany) into 20 μm thick slices. Next, spheroid slices were permeabilized with 0.3% Triton X-100 (T9284, Sigma-Aldrich, BioXtra, USA) and blocked in 2% bovine serum albumin (Sigma-Aldrich, USA). Immunostaining was performed using the following primary antibodies: Ki67 (MAB4190, Merck Millipore, USA), Nestin (MAB5326, Merck Millipore, USA), Beta III-tubulin (MAB1637, Merck Millipore, USA), and Pan-Nav (ab24820, Abcam, USA), all at 1:100 dilutions. The secondary antibodies used were goat antirabbit Alexa Fluor 488 (A11008, Thermo Fisher Scientific, USA) and goat antimouse Alexa Fluor 546 (A11032, Thermo Fisher Scientific, USA), both at 1:400 dilutions. DAPI was used for nuclei counterstaining. Images were acquired using a high-throughput imaging system (Operetta, PerkinElmer, USA).

### 2.3 Detection of Transcripts by PCR

RNA was isolated from cultured cells using the GeneJet RNA extraction kit (Thermo Fisher Scientific, USA) following the manufacturer’s instructions, and subjected to DNase treatment (DNase I, Thermo Fisher Scientific, USA). cDNA was generated from 1 μg of DNase-treated RNA using M-MLV Reverse Transcriptase (Thermo Fisher Scientific, USA). PCR reactions were performed in 10 μL volume using 0.5 μL of input cDNA, 1.5 mM MgCl_2_, 0.2 mM dNTPs, 0.2 μM of each primer and 1 U of Taq Platinum (Thermo Fisher Scientific, USA). Cycling conditions varied according to PCR product size and primers’ melting temperature, but all reactions shared a common initial denaturation step of 95 °C for 3 min, followed by 35 cycles of denaturation at 95 °C for 15 s, annealing at primer specific temperature for 15 s, and elongating at 72 °C for amplicon-specific time. All specific conditions are detailed in Table S1.

### 2.4 Multi-elemental Quantification and Mapping

Multi-elemental analyses were performed in 14 days old neural induced spheroids. Samples were placed on ultralene film (SPEX SamplePrep, USA), quickly rinsed in saline solution and allowed to air-dry. Synchrotron Radiation X-ray Fluorescence (SR-XRF) analyses were performed at former UVX D09B X-ray Fluorescence beamline at the Brazilian Synchrotron Light Source (Campinas, Brazil) using standard temperature and pressure. Samples were excited by a white beam with energy ranging from 5 to 17 keV. An optical system based on a pair of bent mirrors in a Kirkpatrick-Baez arrangement was used to focus the X-ray beam down to approximately 20 μm spatial resolution. Each spot was irradiated for 1 s. A silicon drift detector (KETEK GmbH, Germany) with 140 eV (fwhm) at 5.9 keV placed at 90° from the incident beam was used to collect X-ray fluorescent and scattered radiation coming from samples. Elemental concentration was expressed in weight fraction units determined by the PyMCA software, which was developed by the Software Group of the European Synchrotron Radiation Facility.^20^ Calibration was performed with a set of pure thin films from Micromatter standards (Micromatter, Canada) and the fundamental parameter method was applied.^21^ The weight fraction units were converted in ppm (parts per million) by multiplying them by a 10^6^ factor. Mean concentration value was determined as an average of all irradiated spots.

### 2.5 Statistical Analysis

Analyses of statistical significance were obtained using GraphPad Prism 5 software (GraphPad Software, USA), and unpaired Student’s T-tests were applied to SR-XRF data.

## 3 Results

### 3.1 Characterization of Neural-Induced Spheroids from Dravet iPS Cells

Neural-induced spheroids were generated from control and DS iPS cells. Neural differentiation was confirmed by the detection of nestin and beta III-tubulin transcripts (Figure 1A) and by immunofluorescence staining in all neural-induced spheroids (Figure 1C). Both control and DRVT neural cells exhibited Nav1.1 transcripts, with variable band intensity (Figure 1A) and stained for all Nav isoforms (Pan-Nav; Figure 1B). Channels were regionally distributed within control and DRVT neural cells, presenting a punctate pattern, and Nav distribution at the spheroid level was similar between control and DS-derived cells.

**Figure 1:**
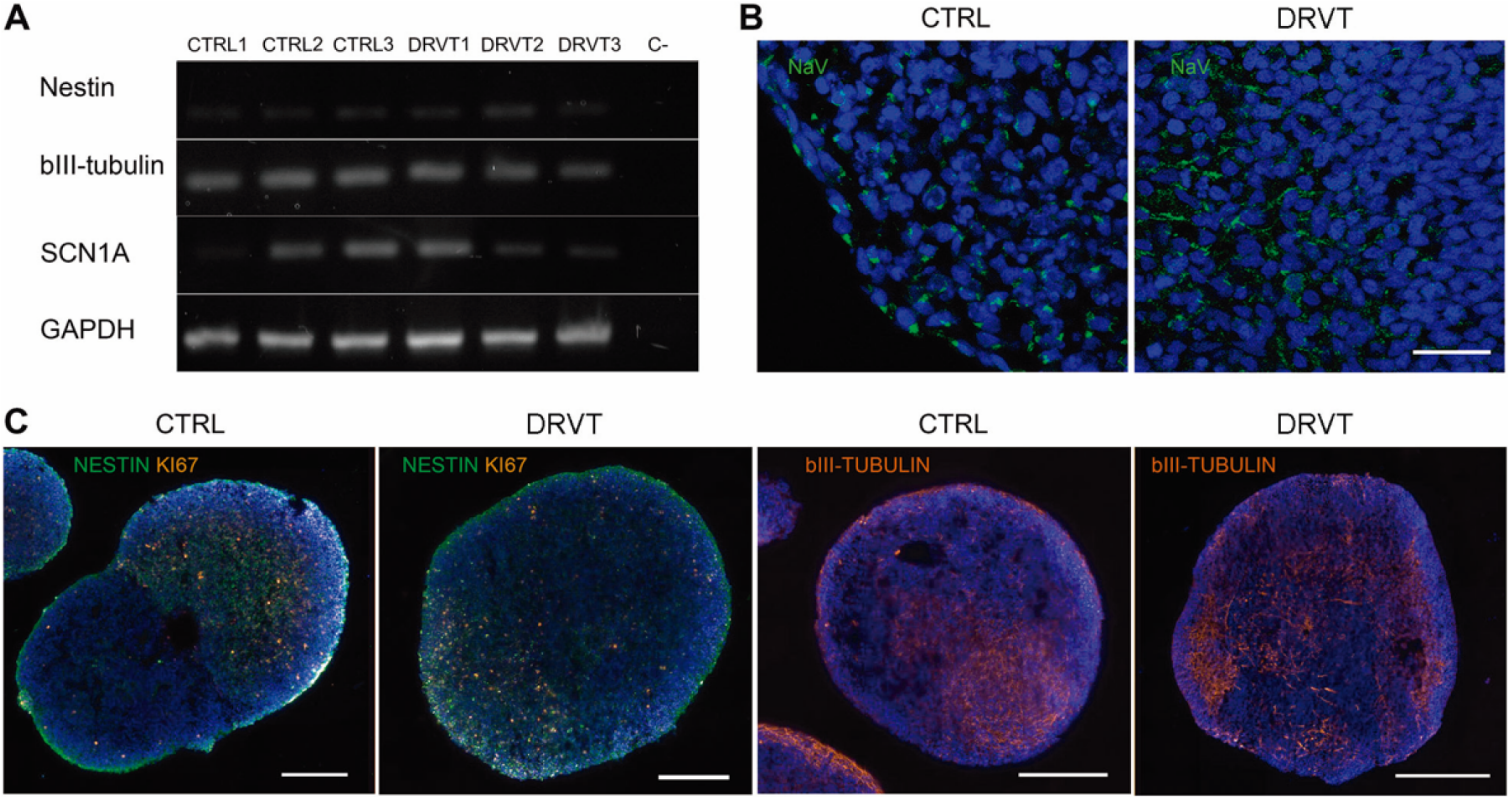
Neural induced spheroids differentiated from Dravet syndrome iPS cells present neural markers and Nav expression. (A) RT-PCR analysis of transcripts of the neuronal markers nestin and class III beta-tubulin (βIII-tubulin) and of the gene encoding neuronal sodium channel, SCN1A. GAPDH was used as internal control. (B) Immunofluorescence detection of all Nav isoforms (Pan-Nav—green) in control (CTRL) and Dravet-derived (DRVT) neural induced spheroids. DAPI shows nuclei counterstaining in blue. Scale bar: 25 μm. (C) Immunofluorescence detection of neuronal cells by nestin (green) and class III beta-tubulin (orange) staining, and for proliferating cells with Ki67 (orange). DAPI shows nuclei counterstaining in blue. Scale bars: 200 μm.

### 3.2 Multi-elemental Characterization of Neural Induced Spheroids

Next, we asked whether there would be elemental differences between control and DRVT cells expressing Nav1.1. To investigate elemental disturbances in these neural cells, samples were collected and analyzed by X-ray fluorescence. Figure 2 shows that DRVT neural induced spheroids presented enhanced levels of potassium (Figure 2A), copper (Figure 2B), and zinc (Figure 2C). Quantification of all elements within detection range is depicted in Table 1.

**Table 1:** Multi-Elemental Quantification of Control and Dravet-Derived Neural Spheroids

Element	Control (PPM) *N*=13	DRVT (PPM) *N*=15	*p* value **p < 0.01
P	223.9 ± 23.7	266.0 ± 15.8	0.1419
S	164.2 ± 40.0	123.6 ± 6.3	0.2925
K	841.9 ± 177.4	1408.0 ± 143.5	**0.0188
Ca	27.0 ± 10.5	27.2 ± 4.1	0.9809
Mn	0.70 ± 0.08	0.70 ± 0.02	0.9554
Fe	3.8 ± 0.9	4.5 ± 0.4	0.4939
Cu	1.5 ± 0.3	3.2 ± 0.3	**0.0012
Zn	10.6 ± 1.7	17.7 ± 1.9	**0.0099

**Figure 2:**
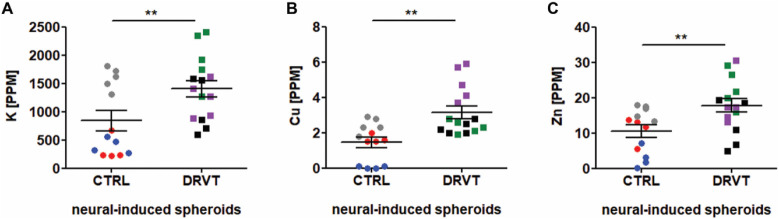
Neural induced spheroids differentiated from Dravet syndrome iPS cells exhibit elevated levels of potassium, copper and zinc. Average element levels (in parts per million—PPM) detected by SR-XRF: (A) Potassium—K. (B) Copper—Cu. (C) Zinc—Zn. Cell lines are represented as CTRL1 (red circles), CTRL2 (blue circles), CTRL3 (gray circles), DRVT1 (green squares), DRVT2 (purple squares), and DRVT3 (black squares). Statistical analyses—Student’s T-test; **p < 0.01.

Although there were differences in the amounts of K, Cu and Zn, the spatial distribution of these chemical elements within neural induced spheroids from Dravet iPS cells and control iPS cells were indistinguishable (data not shown).

## 4 Discussion

Dravet syndrome (DS) has been attributed mostly to de novo mutations in the SCN1A gene.^22^ This gene encodes the α-subunit of the voltage-gated sodium ion channel type 1 (Nav1.1), which contains the voltage sensors and the ion-conducting pore. Most of the known alterations are missense mutations leading to channel loss-of-function.^23^ Perturbations in sodium currents are expected to affect related ions and cellular processes within neurons expressing the mutated channel. Our work is aimed at exploring elemental content and distribution within DS-derived cells, especially neural progenitors and mature neurons, using neural induced spheroids.

Nav1.1 is found in cardiac myocytes^24,25^ and in the central nervous system, particularly, in the cell body and dendrites of GABAergic interneurons.^5–7^ Studies have been done to investigate whether mutated Nav1.1 affects heart tissue;^26–28^ however, DS has been widely observed as a severe epilepsy syndrome. A state of hyperexcitability is thought to arise from diminished firing from GABAergic inhibitory neurons. Symptoms and mechanisms of DS have been well modeled and studied in mice,^7,29,30^ and more recently, in iPS-derived neural cells;^31,32^ however, published data have shown controversial evidence regarding the importance of Nav1.1 in inhibitory and excitatory neurons.^33,34^ When studying DS, it is important to take into consideration the elevated number of already described SCN1A mutations, as different alterations can lead to slightly different outcomes.^35^ Cell reprogramming allows the generation of patient-specific neural cells that may recapitulate some disease mechanisms. Our data show that DS and control cells are fully reprogrammed and able to differentiate into all three germ layers, forming EBs, and, with adequate stimulus, these EBs can be enriched in neural cells, providing a fast and practical method for evaluating DS cell behavior. Although the literature indicates that Nav1.1 is predominantly expressed after birth,^36,37^ we were able to detect SCN1A transcripts and Nav1.1 protein in 14 days old neural induced spheroids from control and DS cells. These characterizations provide insights into the use of the iPS differentiation model in vitro to study DS; however, additional approaches may also contribute to the field. Electrophysiology analyses of DS-derived neural induced EBs, for instance, may allow the observation of an altered firing pattern compared to control EBs.^38^

Next, in order to further understand the impact of SCN1A mutation and its resulting nonfunctional channel on a systemic level, we analyzed neural induced spheroids by SR-XRF.^15–17^ Performed at the former Brazilian light source, UVX (LNLS), this technique provides highly sensitive multi-elemental quantification and mapping of cellular aggregates. Our analyses have revealed that iPS cells differentiated into 14 days old neural cells spheroids present elevated levels of potassium (K), copper (Cu) and zinc (Zn). Other elements, such as calcium and iron, which were also detected, did not, however, present differences between control and DS cells (Table 1).

Although our model of neural induced spheroids represents a very early stage of neural development, we have been able to detect SCN1A transcripts, Nav expression and changes in elemental balance that may, in part, represent neuronal (and related cells) impaired activity in DS patients. Differences in elements’ levels may be the cause or consequence of elemental equilibrium in DS, yet, in order to understand the role of K, Cu and Zn imbalance in the disease, we focused on the possible consequences of the reduced function of Nav1.1 channels.

The X-ray microfluorescence approach used in this work allows the distinction between the elemental composition of cell clusters and background, however, it does not reach resolution to distinguish between an increase in extracellular or intracellular potassium. Ion levels in neural tissue are tightly regulated;^39^ therefore, the changes found in our in vitro model are likely due to the differences found in the activity of DS-derived cells compared to control cells.

Nevertheless, one could hypothesize that reduced sodium inward currents due to Nav1.1 loss-of-function might affect potassium distribution as these cations are tightly and closely regulated. If less sodium enters the cell that expresses the mutated Nav1.1, we hypothesize that its Na^+^/K^+^ ATPase activity might be reduced, causing potassium to be released from local hyperexcitable neurons to accumulate in the extracellular space (Figure 3A). Spheroids and embryoid bodies present internal organization with the expression of extracellular matrix components,^40,41^ possibly creating a microenvironment able to retain extracellular potassium. Excess extracellular potassium by itself could directly augment excitability of surrounding neurons by stimulating membrane depolarization.^42,43^ Animal models have led to the idea that Nav1.1 is predominant in GABAergic neurons, which by being less frequently depolarized in the absence of this channel would release less GABA and therefore decrease inhibition of excitability, making the brain more susceptible to seizures.^5–7^ However, this hypothesis was not entirely confirmed in human cellular models.^41,44–46^ Here we show evidence that altered sodium channels cause an imbalance in potassium concentration that might also explain, at least partially, the increased excitability associated with this disease.

**Figure 3:**
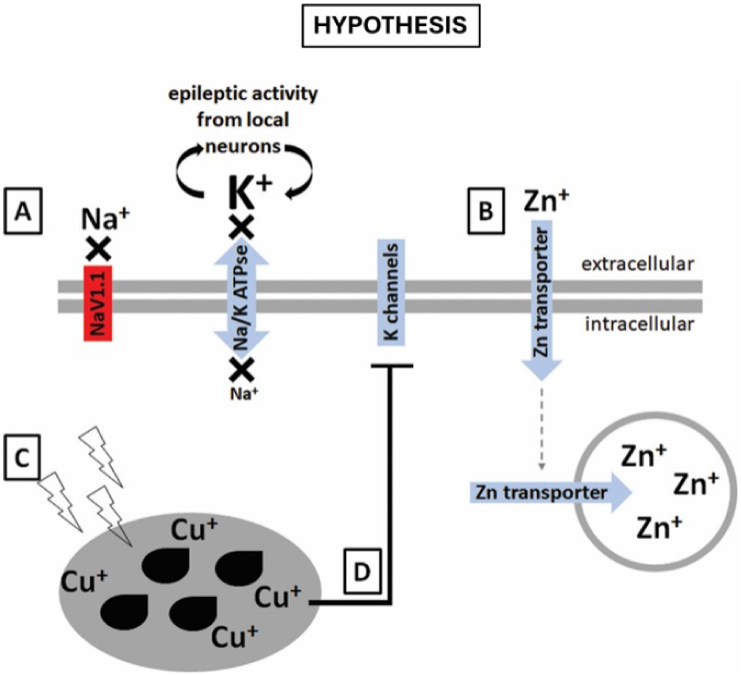
Hypothetical model for the elemental dynamics in Dravet syndrome neural cells. Our hypothesis for the findings involves Nav1.1 channels in DRVT neural cells not functioning properly, with neurons not able to fire action potentials, leading to (A) potassium extracellular accumulation by excessive action potential firing from neighboring neurons and reduced Na/K ATPase clearing activity. As neurons do not fire action potentials, (B) zinc presynaptic vesicles accumulate. Finally, intensive oxidative stress generated by the hyperexcitability state derived from low GABAergic activity may lead to elevated levels of (C) copper-dependent antioxidative enzymes. Copper elevated levels further inhibit potassium channels (D), contributing to Na/K ATPase reduced activity and extracellular potassium accumulation.

Zinc is also a very relevant trace element in neural cells. Indeed, alterations in zinc levels lead to neuronal damage and death.^38^ Although presynaptic zinc is mostly found in glutamatergic neurons, it has been found in colocalization with GAD (glutamic acid decarboxylase) and ZnT3 (zinc transporter-3) in GABAergic neurons of mouse spinal cord.^47^ We hypothesize that DS neural cells may express ZnT3 transporter, which allows constant zinc transport from the extracellular space into the cytoplasm, and from the cytoplasm into vesicles.^40,41^ However, we also hypothesize that impaired ionic balance in DS neural cells, may let zinc accumulate into presynaptic vesicles that will seldom be released into the extracellular space, culminating in overall elevated zinc levels (Figure 3B). It is important to note that Zn is also an inhibitor of GABA and NMDA receptors;^42–45^ therefore, even in the rare event of Zn vesicles release, this element could reinforce the hyperexcitable state found in DS patients. Future studies on zinc-filled vesicles release activity in DS neural cells will contribute to further elucidating this matter.

Copper is profoundly involved in brain development and function. As a cofactor of several redox enzymes, it is often associated with oxidative stress.^48^ Free radicals are frequently generated during seizures, and the elevated levels of copper may correlate with both oxidative damage itself and the antioxidative response in epilepsies, mainly via SOD1.^47–49^ Although reports show that SOD1 is downregulated in epilepsy,^50^ there is evidence of SOD1 upregulation in DS iPS neural cells under specific circumstances, such as incubation with human umbilical cord mesenchymal stem cells-conditioned medium.^51^ In this work we hypothesize that a highly oxidative environment might trigger SOD1 expression, increasing local SOD1-associated copper because of the cells’ antioxidant response. If SOD1 expression is elevated in DS neural cells, copper concentration might reflect that change (Figure 3C) and future studies on SOD1 expression in DS neural cells might shed light in the presence of high copper concentrations in these cells. Reports on copper serum levels in epilepsy patients reveal that the use of anticonvulsant drugs, such as phenobarbital, might mask actual copper levels in brain tissue.^52^ Therefore, data on DS should be interpreted taking into consideration that studies involving patients under treatment and iPS-based studies might present divergences when considering micronutrients’ bioavailability. In addition, copper has an inhibitory effect on GABA receptors and on A-type potassium channels,^53^ which might contribute to further explaining potassium elevated levels found in DS neural induced EBs (Figure 3D). Studies on potassium transport in DS neural cells might elucidate these networks.

We conclude that modeling DS using cell reprogramming and spontaneous and/or directed neural differentiation are relevant approaches to understand the mechanisms involved in this disease and, most importantly, to develop in vitro platforms for the testing of candidate treatment molecules. To date, this is the first report on the elemental composition of Dravet-derived cells, and, although a cell-based study has its limitations, further studies might point toward the reestablishment of K, Cu and Zn levels, as a possible strategy to affect DS neural cells and, possibly, find an alternative way of treating and controlling this syndrome.

## Supplementary Material



## Notes

Table S1: Primer sequences (PDF)
