# Supplementary material for: Dravet Syndrome Patient-Derived Neural Cells Present Altered Levels of Potassium, Copper, and Zinc
Source: ACS Chem Neurosci. 2025 Dec 24;17(1):308–14. doi: 10.1021/acschemneuro.5c00898 (PMC12784395; doi:10.1021/acschemneuro.5c00898)
Supplement: Supplementary file 1 [file cn5c00898_si_001.pdf]

Supplemental table 1: Primer sequences

Primer	Sequence 5'-3'	Amplicon size	Annealing temp.	Elongation time
**GAPDH-F**	TTC GAC AGT CAG CCG CAT C	352 bp	58°C	13 s
**GAPDH-R**	GAC TCC ACG ACG TAC TCA GC			
**hBTub-F**	TGC AGC AGC TGG ACA ATG	117 bp	60°C	10 s
**hBTub-R**	AGC GCA GTG CGT AGA AGA TG			
**hNestin-F**	AGC CCT GAC CAC TCC AGT TTA G	128 bp	60°C	10 s
**hNestin-R**	CCC TCT ATG GCT GTT TCT TTC TCT			
**hSCN1A-F**	AAC AGA ATC AGG CCA CCT TG	141 bp	60°C	10 s
**hSCN1A-R**	CAC TGG GCT CTC TGG AAT G			
